# Cost-Effectiveness of Fetal Fibronectin Testing in Women at Risk for Preterm Birth: A Retrospective Cohort Study

**DOI:** 10.2147/CEOR.S611886

**Published:** 2026-08-08

**Authors:** Dina Abushanab, Nader Al-Dewik, Reem AlNasr, Reem Alenany, Lana Kattan, Ghaith Alali, Alaa Al-Naama, Moza Al-Hail, Thomas Farrell, Haseebur Rahman Khan Mohammed, Wessam Elkassem, Abdulrouf Pallivalapila, Binny Thomas, Daoud Al-Badriyeh

**Affiliations:** 1 Office of Vice President for Health and Medical Sciences, QU Health, Qatar University, Doha, Qatar; 2 Department of Research, Women’s Wellness and Research Center, Hamad Medical Corporation, Doha, Qatar; 3 Department of Pediatrics and Neonatology, Neonatal Intensive Care Unit, Newborn Screening Unit, Women’s Wellness and Research Center, Hamad Medical Corporation, Doha, Qatar; 4 Translational and Precision Medicine Research and Interim Translational Research Institute (Itri), Hamad Medical Corporation, Doha, Qatar; 5 Faculty of Health and Social Care Sciences, Kingston University and St. George’s University of London, London, UK; 6 Genomics and Precision Medicine, College of Health and Life Science, Hamad Bin Khalifa University, Doha, Qatar; 7 College of Pharmacy, QU Health, Qatar University, Doha, Qatar; 8 Obstetrics and Gynecology Department, Women’s Wellness and Research Center, Hamad Medical Corporation, Doha, Qatar; 9 Pharmacy Department, Hamad Medical Corporation, Doha, Qatar; 10 Clinical Trial Unit, Hamad Medical Corporation, Doha, Qatar

**Keywords:** cost-effectiveness analysis, fetal fibronectin, preterm birth, preterm delivery, pregnancy

## Abstract

**Introduction:**

The fetal fibronectin (fFN) test is used to predict true preterm delivery. This study aims to evaluate the cost-effectiveness of fFN testing versus no-fFN testing in identifying true preterm labor among women with preterm labor symptoms at risk for premature birth.

**Methods:**

This retrospective cohort-based study was conducted among women who presented with symptoms of preterm labor between 24 and 34 weeks of gestation at a tertiary care hospital in Qatar and compared outcomes between those who received fetal fibronectin (fFN) testing and those who did not. A decision-tree model compared fFN testing to no testing, with the primary outcome being the incremental cost-effectiveness ratio, representing the additional cost for a successful delivery within 48 hours of admission. Clinical data were collected from HMC medical records, and direct medical costs was locally obtained, reflecting the Qatari hospital perspective. Sensitivity analyses confirmed the study results.

**Results:**

A total of 519 women were analyzed. Significant baseline differences were observed between groups. The proportion of delivery within 48 hours was higher in the fFN group (94% vs 80%, P<0.0001). fFN testing was associated with a cost saving of QAR6,470 (US$1,777) per successful delivery.

**Conclusion:**

fFN testing was associated with better short-term clinical outcomes and lower healthcare costs. Nonetheless, due to the retrospective observational nature of the study and differences in baseline characteristics between groups, these results should be interpreted cautiously.

## Introduction

Preterm labor is a clinical condition that can arise from various underlying causes, potentially leading to preterm birth. It is a major global health problem, affecting approximately 5–13% of deliveries worldwide.^1^ Preterm birth, delivery before 37 weeks of gestation, is a major contributor to neonatal illness and death. Women presenting with preterm labor between 24 and 34 weeks of gestation are 2–3 times more likely to deliver preterm.^2^ This condition also contributes to substantial healthcare costs, estimated in the United States (US), as example, at US$ 25 billion annually.^3^

Studies have shown that 75–95% of women with preterm labor do not deliver within seven days, and approximately 40% ultimately deliver at term.^4^,^5^ Furthermore, 44% of these women experience subsequent admissions for preterm labor, leading to increased healthcare costs.^5^ Accurate risk stratification in women who present with symptoms of preterm labor is therefore essential to inform decisions about hospitalization, corticosteroid use, tocolytic treatment, and transfer to higher-acuity care, while minimizing unnecessary interventions and reducing healthcare costs. Thus, early identification of preterm labor is important for mitigating neonatal and maternal risks and financial burdens.

Multiple strategies have been developed to enhance risk stratification in women with symptoms of preterm labor. These include measuring cervical length and performing fetal fibronectin (fFN) testing, both of which help estimate the probability of imminent preterm birth and inform clinical management.^1^,^6–16^ Recent evidence continues to reinforce the use of biomarker-based approaches and cervical assessment strategies for risk stratifying women with symptoms of preterm labor.^17^,^18^ Cervical length measurement reflects structural changes such as cervical shortening, while fFN testing detects a biochemical marker indicative of disruption at the maternal–fetal interface. Nonetheless, no single approach has proven consistently superior, and guideline recommendations for their use differ.^19^,^20^ One major benefit of fFN testing is its high negative predictive value; a negative result strongly indicates a low chance of delivery in the following days. This can help clinicians reduce unnecessary hospital admissions and treatments for women unlikely to give birth soon.^21^ Still, the test has drawbacks: positive results can be affected by multiple clinical factors and do not always correspond to imminent preterm delivery.^22^

Studies from Europe and North America have examined the clinical and economic impact of fFN testing and other risk-stratification methods for threatened preterm labor.^8^,^23–25^ Although several studies suggest possible decreases in unnecessary admissions and healthcare expenses, the results differ based on local healthcare environments, clinical management pathways, and patterns of resource use. As a result, it is unclear how well findings on cost-effectiveness apply across different healthcare systems.

Although fFN testing is used routinely in Qatar, there is limited local evidence on its cost-effectiveness and its effect on healthcare resource use in the Qatari healthcare system. Because healthcare delivery, admission practices, treatment approaches, and costs vary between countries, country-specific economic evaluations are necessary to inform local clinical care and resource-allocation decisions. At the Women’s Wellness and Research Center (WWRC) of Hamad Medical Corporation (HMC),^26^ fFN testing has been used since 2019 to identify cases of suspected preterm labor. However, there is currently a lack of evidence to justify, to decision-makers, the resource allocation for fFN testing. This is particularly important in light of recent developments related to the availability of fFN test. While Hologic’s decision to cease production of fFN cassettes creates challenges in the United Kingdom (UK),^27^ it highlights the need to evaluate its cost-effectiveness in settings where the test remains accessible, such as in Qatar. Here, economic evaluations are indeed needed to guide the economic versus benefit consequences associated with this testing. Therefore, this study aims to evaluate the clinical and economic outcomes associated with fFN testing compared to no-fFN testing among women presenting with preterm labor symptoms at the WWRC of HMC in Qatar.

## Methods

### Ethics Approval

Ethics approval was obtained from the Medical Research Center at HMC, Qatar, for data access under protocol MRC-01-23-108. The need for informed consent to participate was waived by an Institutional Review Board of the MRC at HMC because the study was retrospective, relied on existing medical records, presented minimal risk to participants, and had no impact on patient care or clinical management. To ensure patient confidentiality, all data were de-identified before analysis, and no personally identifiable information was collected or reported. Access to the study dataset was limited to authorized study investigators and handled according to HMC data protection policies and relevant ethical requirements. This study was conducted in full conformance with principles of the “Declaration of Helsinki”, Good Clinical Practice (GCP) and within the laws and regulations of Ministry of Public Health in Qatar.

### Study Design and Setting

This study was a retrospective cohort-based cost-effectiveness analysis conducted at the WWRC of HMC in Qatar. HMC is the principal public healthcare provider in the country, comprising fourteen hospitals, with the WWRC being the largest tertiary referral public center for women’s and neonatal health, equipped with 378 beds.^26^ The analysis included women with preterm labor admitted to the labor and delivery unit of WWRC.

### Population

The study population included women with established preterm labor, defined as those experiencing regular, painful uterine contractions occurring at least once every ten minutes, with ruptured fetal membranes and/or documented cervical change. For primigravida women, the criteria include a fully effaced cervix or dilation greater than 2 cm, while, for multigravida women, the cervical criteria include full effacement or dilation greater than 3 cm.

#### Inclusion Criteria

- Women with preterm labor between 24–34 weeks of gestational age, confirmed by ultrasound and the date of their last menstrual period.
- Women who underwent fFN testing during the study period.
- Women who did not undergo fFN testing during the study period.

#### Exclusion Criteria

- Women with active vaginal bleeding, cerclage, clinical signs of abruption, preterm labor occurring after 34 weeks of gestational age, systemic infection, or fetal abnormalities.

### Sample Size Calculation

There are currently no reported head-to-head studies that compare the delivery of preterm labor, within 48 hours, between the use of fFN testing and the absence of fFN testing. Due to the retrospective design, the sample size was chosen pragmatically based on the number of eligible patients available and on sample sizes reported in prior studies assessing fFN testing in comparable populations. A review of relevant literature indicates that the number of women included in these studies can range from fewer than 100 to fewer than 1,000.^8^,^23–25^ Considering this range, we have established a target sample size of a minimum of 500 patients combined for both groups in our analysis. We anticipated that a three-year data collection period (2019–2022) will allow us to achieve this target, providing a robust foundation for investigating the effects of fFN use versus no-fFN testing in our study setting.

To select participants for the study groups, patient medical records were retrieved in descending order of hospital admission numbers from the Cerner electronic medical records database. Women were screened for inclusion until the target sample size was reached or until no further patient records were available. Any excluded patient records, including due to missing data, were replaced by ordering additional records if they were available.

### Model Overview

A cost-effectiveness analysis, from the HMC hospital perspective, compared the use of fFN testing against no-fFN testing. All women experiencing preterm labor were monitored until their hospital discharge (ie, general ward discharge). The economic evaluation was performed in accordance with established health economic principles and CHEERS 2022 reporting standards.^28^

**Figure 1:**
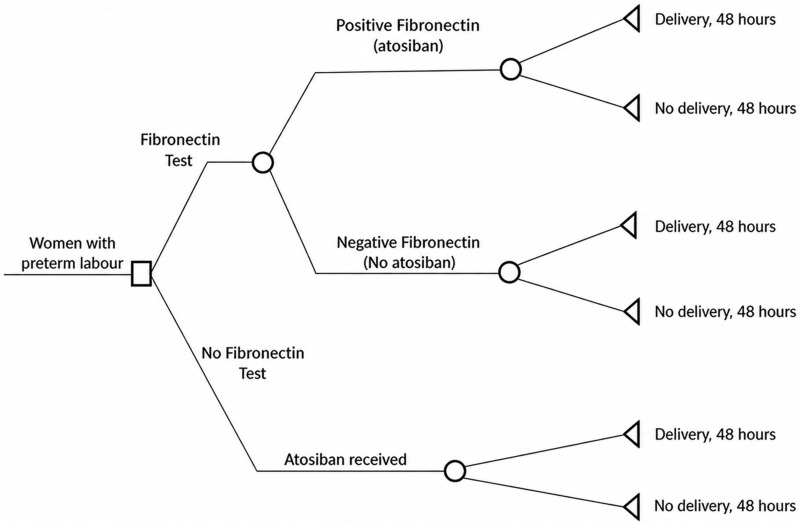
Decision-analytic model tree of study groups.

The economic evaluation was based on a conventional decision-tree model to assess the cost-effectiveness of achieving delivery outcomes. The model constitutes six patient pathways, starting with categorizing women into one of two exclusive groups: fFN testing and no-fFN testing. The model adopted health outcomes identified from a previously published decision tree model,^8^ as well as input from clinicians involved in our study. When the fFN was positive, patients received one cycle of atosiban and antenatal corticosteroids. When the fFN was negative, no atosiban and antenatal corticosteroids were administered. Of note, in the fFN arm, probabilities were modeled conditionally based on test outcome (positive vs negative), while in the no-fFN arm, probabilities reflect the overall observed cohort. A decision-tree model was used because the study examined short-term clinical and economic effects within a limited time horizon. The model captured the main clinical pathways and management choices for women with symptoms of preterm labor, including fFN testing, treatment decisions, and delivery outcomes occurring within 48 hours. The model tree is illustrated in Figure 1. Model probabilities used in the decision tree were reported in the results and were not displayed within Figure 1 for clarity of structure.

### Model Inputs

Clinical inputs, including demographic and probability data, were retrospectively extracted from HMC’s Cerner medical records database for the period of 2019 to 2022. Baseline variables were chosen based on clinical relevance and availability within the electronic medical records, including demographic characteristics, obstetric history, and clinical presentation at admission.

For the cost model inputs, and given the HMC hospital perspective of the study, the analysis was performed from the healthcare provider perspective and included only direct medical costs. Costs were calculated using a micro-costing approach based on hospital charges, with resource utilization data obtained from the electronic medical records at HMC. Indirect costs, productivity losses, caregiver burden, and other societal costs were not captured. The resources included in the analysis are:

- fFN testing.
- Medications, eg, tocolytics, antibiotics, corticosteroids.
- Diagnostics, laboratory, and monitoring tests performed during hospitalization.
- Management of adverse drug events (ADEs).
- Hospital stay, excluding other resource costs.

The administration of one cycle of atosiban started from the initial presentation in the emergency department. The atosiban tocolytic cycle consists of an initial dose of 6.75 mg administered as a slow intravenous bolus over 1 minute, followed by a continuous infusion of 300 mcg/minute for 3 hours, and then a reduced infusion rate of 100 mcg/minute for up to 45 hours (with a maximum total dose of 330.75 mg over 48 hours).^29^

All costs were calculated in Qatari Riyal (QAR) and adjusted for the financial year 2024–25 using the Qatari Health Consumer Price Index. The cost-effectiveness analysis focused on successful outcomes, both and without ADEs. No discounting was applied, as outcomes were not projected beyond a one-year time horizon.

### Outcomes

#### Primary Measures

- The incremental cost-effectiveness ratio (ICER), defined as the added cost per additional case of successful delivery within 48 hours. The latter refers to the successful prolongation of delivery within 48 hours. Success delivery was defined as so regardless of ADEs occurrence. This 48-hour period is particularly significant because it reflects an important window where effective intervention can lead to improved clinical outcomes for both the woman and the neonate.

#### Secondary Measures

- Delivery >48 hours and ≤7 days, occurring more than 48 hours but less than or equal to 7 days after receiving atosiban.
- Delivery >7 days and ≤14 days, occurring more than 7 days but less than or equal to 14 days after receiving atosiban.
- Time from admission to delivery, measured in days.
- Length of hospital stay, defined as the duration from admission to discharge.
- Hospital readmission, occurring within 14 days for women with initial negative fFN results.
- All-cause mortality evaluated during hospitalization.
- ADEs associated with atosiban administration, including tachycardia, palpitations, vomiting, nausea, hypotension, headache, injection site reactions, and tremors.^29^
- Admission of preterm infants to the neonatal intensive care unit (NICU).
- Neonatal comorbidities, including respiratory distress syndrome (RDS), bronchopulmonary dysplasia (BPD), early and late sepsis, patent ductus arteriosus (PDA), periventricular leukomalacia (PVL), intraventricular hemorrhage (IVH), necrotizing enterocolitis (NEC), and retinopathy of prematurity (ROP).
- Neonatal deaths, defined as deaths among live births during the first 28 completed days of life.^30^

### Statistical Analysis

Both descriptive and inferential analyses were conducted, using frequencies and percentages for clinical categorical data, and means ± standard deviations (SD) or medians ± interquartile ranges (IQR) for clinical continuous variables. Statistical differences in baseline characteristics and clinical outcomes between the fFN and no-fFN groups were assessed using independent t-tests, Mann–Whitney *U*-tests, Chi-square tests, or Fisher’s exact tests as appropriate. A 95% CI and 80% power were considered to determine statistical significance. Data were analyzed using the Statistical Package for the Social Sciences (SPSS^®^) version 28. Data completeness was evaluated before the analysis. As there were no missing values for any variables included in the study, no imputation procedures were necessary. Because this was a retrospective observational study, the goal was to detect associations rather than prove causation.

### Sensitivity Analysis of Economic Model

Parameter uncertainty was investigated through one-way sensitivity analyses (OWSA), in which one parameter was varied at a time to identify the parameters with the greatest impact on results.^31^ Additionally, a multivariate sensitivity analysis (MSA) was conducted, where all variables were altered simultaneously.^31^

For the OWSA, we varied the cost of fFN testing, setting the upper and lower bounds at the 95% confidence interval (CI) using a gamma input distribution. In the MSA, all the probability and cost model inputs were varied, considering beta distribution for probability inputs and gamma distribution for cost inputs, also based on the 95% CI. The results of MSA sensitivity analysis were presented as cost-effectiveness acceptability curves (CEACs) and a Tornado diagram to demonstrate the extent to which the base-case ICER is sensitive to changes in underlying parameters.^31^ All sensitivity analyses were performed using Monte Carlo simulation, at 10,000 iterations, via @Risk- 7.5^®^, Palisade Corporation, NY, USA.

The model adhered to the reporting followed the Consolidated Health Economic Evaluation Reporting Standards 2022 (CHEERS 2022) (Supplementary Table 1).

## Results

### Demographic Characteristics of Women

A total of 519 women were included in this analysis, with 232 in the fFN group and 287 in the no-fFN group. There were multiple baseline clinical differences between the groups, such as membrane rupture and gestational age at previous admission, indicating the groups may not be directly comparable.

The fFN group demonstrated a slightly higher median maternal age (31 vs 30 years, P=0.022). Despite this difference, both groups were comparable in terms of basic anthropometric measurements, with no significant differences observed in BMI (27.73 vs 28.41 kg/m^2^, P=0.383), weight (P=0.143), or height (P=0.338). Similarly, obstetric history parameters, including gravidity (P=0.274) and parity (P=0.147), were not significantly different between the groups. Women in the fFN group had markedly different gestational timelines, with a median gestational age of 29 weeks at previous admission compared to 0 weeks in the no-fFN group (P<0.001). This trend continued to delivery, with the fFN group reaching a later gestational age at birth (37 vs 35 weeks, P<0.001). Furthermore, the distribution of multiple pregnancies was similar between groups (P=0.167), with most being singleton pregnancies in both groups (90.5% vs 85.3%, respectively).

The mode of delivery differed significantly between groups (P<0.001), with the fFN group showing a higher rate of normal vaginal delivery (48.3% vs 39.4%). The fFN group also experienced fewer complications, with significantly lower rates of rupture of membrane on admission (5.2% vs 35.5%, P<0.001) and intrapartum bleeding (1.7% vs 7.0%, P=0.005). Despite the higher rates of suspected chorioamnionitis in the fFN group (6.5% vs 2.8%, P=0.043), the fFN group required less antibiotic administration (63.9% vs 73.5%, P=0.019). Other interventions, including tocolytic administration during initial admission, steroid use, and blood transfusion requirements were comparable between groups. A comprehensive description of the maternal characteristics can be found in Table 1.

**Table 1:** Main Baseline Maternal Demographic Characteristics

Characteristic	Fibronectin (n=232)	No Fibronectin (n=287)	*P value*
Maternal age, median (IQR), years	31 (8)	30 (9)	0.022
Gestational age at previous admission, median (IQR), weeks	29 (4)	0 (24)	<0.001
Gestational age at birth, median (IQR), weeks	37 (3)	35 (3)	<0.001
Weight, median (IQR)	70 (19.8)	72 (21)	0.143
Height, median (IQR)	158 (9)	158 (7.7)	0.338
BMI, median (IQR), kg/m^2^	27.73 (7.6)	28.41 (8.03)	0.383
Gravida, median (IQR)	3 (2)	3 (3)	0.274
Parity, median (IQR)	1 (3)	1 (3)	0.147
Cervical length, median (IQR), cm	0 (3.10)	0 (0)	<0.001
Cervical dilation, no. (%), cm			0.098
0–3 cm	135 (72.2)	137 (74.9)	
4–7 cm	49 (26.2)	37 (20.2)	
8–10 cm	3 (1.6)	9 (4.9)	
Nationality, no. (%)			0.754
Qatari	83 (35.8)	96 (33.4)	
Arab, non-Qatari	77 (33.2)	104 (36.2)	
Non-Arab	72 (31.0)	87 (30.3)	
Number of fetuses, no. (%)			0.167
Singleton	210 (90.5)	244 (85.3)	
Twin	18 (7.8)	37 (12.9)	
Triplets	4 (1.7)	5 (1.7)	
Mode of delivery, no. (%)			<0.001
Normal vaginal	112 (48.3)	113 (39.4)	
Caesarean	97 (41.8)	167 (58.2)	
Vaginal forceps assisted	8 (3.4)	1 (0.3)	
Vaginal vacuum assisted	15 (6.5)	6 (2.1)	
Risk factors for preterm birth, no. (%)			0.083
No risk factors	125 (53.9)	125 (43.6)	
History of cesarean delivery	48 (20.7)	76 (26.5)	
History of premature birth	12 (5.2)	11 (3.8)	
Poly or oligo hydramnios	4 (1.7)	15 (5.2)	
History of premature birth	12 (5.2)	17 (5.9)	
Current conception by IVF	16 (6.9)	18 (6.3)	
History of miscarriage	14 (6.0)	25 (8.7)	
History of uterine surgery	1 (0.4)	0 (0)	
Infection, no. (%)			0.243
No infection	226 (97.4)	282 (98.3)	
UTI	1 (0.4)	4 (1.4)	
Bacterial vaginosis	1 (0.4)	0 (0)	
Syphilis	1 (0.4)	0 (0)	
COVID-19	3 (1.3)	1 (0.3)	
Suspected chorioamnionitis, no. (%)			0.043
Yes	15 (6.5)	8 (2.8)	
No	217 (93.5)	279 (97.2)	
ROM on admission date, no. (%)			<0.001
Yes	12 (5.2)	102 (35.5)	
No	219 (94.8)	185 (64.5)	
Intrapartum bleeding, no. (%)			0.005
Yes	4 (1.7)	20 (7)	
No	228 (98.3)	267 (93)	
Postpartum bleeding, no. (%)			0.758
Yes	11 (4.7)	12 (4.2)	
No	221 (95.3)	275 (95.8)	
Blood transfusion, no. (%)			0.256
Yes	8 (3.5)	16 (5.6)	
No	223 (96.5)	271 (94.4)	
Congenital abnormality, no. (%)			1.000
Yes	0 (0)	1 (0.3)	
No	232 (100)	286 (99.7)	
Tocolytic (Atosiban) received during initial admission (1^st^ full cycle), no. (%)			0.561
Yes	28 (12.1)	30 (10.5)	
No	204 (87.9)	257 (89.5)	
Tocolytic (Atosiban) received during initial admission (2^nd^ cycle), no. (%)			0.119
Yes	28 (12.1)	23 (8.0)	
No	203 (87.9)	264 (92.0)	
Antibiotics received, no. (%)			0.019
Yes	147 (63.9)	211 (73.5)	
No	83 (36.1)	76 (26.5)	
Steroids received, no. (%)			0.483
Yes	106 (45.7)	140 (48.8)	
No	126 (54.3)	147 (51.2)	
Reason for antibiotics, no. (%)			0.181
Caesarean delivery	1 (0.4)	0 (0)	
Chorioamnionitis	7 (3.0)	1 (0.3)	
Fever	1 (0.4)	0 (0)	
GBS positive	42 (18.1)	5 (1.7)	
GBS positive and chorioamnionitis	2 (0.9)	0 (0)	
GBS positive and leaking	1 (0.4)	0 (0)	
GBS positive, leaking, and chorioamnionitis	1 (0.4)	0 (0)	
HBV and leaking	0 (0)	1 (0.3)	
History of sepsis	2 (0.9)	0 (0)	
Leaking	7 (3.0)	9 (3.1)	
Leaking and chorioamnionitis	1 (0.4)	0 (0)	
MRSA positive	1 (0.4)	1 (0.3)	
No antibiotics	85 (36.6)	75 (26.1)	
Preterm	20 (8.6)	15 (5.2)	
Preterm and chorioamnionitis	0 (0)	1 (0.3)	
Preterm and fever	1 (0.4)	0 (0)	
Preterm and GBS positive	2 (0.9)	5 (1.7)	
Preterm and leaking	6 (2.6)	8 (2.8)	
Preterm and MRSA positive	1 (0.4)	0 (0)	
Preterm and pneumonia	0 (0)	1 (0.3)	
Preterm and PROM	2 (0.9)	0 (0)	
Preterm, PROM, and GBS positive	0 (0)	1 (0.3)	
PROM	2 (0.9)	31 (10.8)	
PROM and chorioamnionitis	2 (0.9)	1 (0.3)	
PROM and GBS positive	1 (0.4)	2 (0.7)	
PROM and UTI	0 (0)	2 (0.7)	
Antibiotics prophylaxis	32 (13.8)	38 (13.2)	
Blood pressure status, no. (%)			0.053
Chronic HTN with superimposed preeclampsia	0 (0)	2 (0.7)	
Chronic HTN	5 (2.2)	7 (2.4)	
Gestational HTN	5 (2.2)	12 (4.2)	
None	216 (93.1)	246 (85.7)	
Preeclampsia	6 (2.6)	20 (7.0)	
Glycemic status, no. (%)			0.050
DM type I or II	10 (4.3)	11 (3.8)	
Gestational DM	72 (31.0)	85 (29.6)	
GTT normal	66 (28.4)	66 (23.0)	
No GTT/HbA1C/fasting glucose	24 (10.3)	19 (6.6)	
Normal GTT or HBA1C	59 (25.4)	106 (36.9)	
GBS status, no. (%)			<0.001
Negative	176 (75.9)	268 (93.4)	
Positive	51 (22.0)	19 (6.6)	
Not performed	5 (2.2)	0 (0)	

**Abbreviations**: IQR, interquartile range; BMI, body mass index; no, number; %, percentage; IVF, in vitro fertilization; UTI, urinary tract infection; ROM, rupture of membranes; GBS, group B streptococcus; HBV, hepatitis B virus; MRSA, Methicillin-resistant *Staphylococcus aureus*; PROM, premature rupture of membranes; HTN, hypertension; DM, diabetes mellitus; GTT, glucose tolerance test; HbA1C, hemoglobin A1C.

### Demographic Characteristics of Neonates

Neonatal characteristics showed several significant differences between the groups. Infants born to mothers in the fFN group had notably higher median birth weights (2920g vs 2255g, P<0.001). Both groups showed differences in APGAR score distributions at 1 and 5 minutes (P<0.001 for both time points). Despite these variations, NICU admission rates were similar between the groups (70.3% vs 67.9%, P=0.611). RDS appeared as the predominant neonatal complication in both groups. The fFN group showed 38 cases of RDS compared to 29 cases in the no-fFN group. However, the pattern of RDS complications differed between groups, with the no-fFN group showing a higher tendency for RDS combined with other complications, particularly sepsis (8 cases vs 2 cases in the fFN group). Low birth weight complications were less frequent in the fFN group, aligning with the higher median birth weight observed in this group. Jaundice occurrence was similar between groups, while various other complications occurred at different frequencies. A thorough overview of the neonatal characteristics is provided in Supplementary Table 2.

### Base-Case Analysis

No ICER was calculated in the current analysis due to the identified dominance status, whereby one intervention was associated with a higher outcome and lesser cost than the comparator. Compared to a 94% rate of successful delivery within 48 hours observed in the fFN group, the no-fFN testing group achieved of the outcome with an 80% rate (risk ratio 0.31; 95% CI 0.18–0.55; P<0.0001). This difference was associated with a cost saving of QAR6,470 (US$1,777) per case of successful delivery within 48 hours, regardless of the presence of ADEs, also in favor of the fFN testing. Table 2 presents the outcome probabilities and weighted costs associated with fFN and no-fFN.

**Table 2:** Outcome Probabilities and Weighted Costs of Fibronectin and No Fibronectin (Total Population)

Outcome	Probability	Cost, QAR	Proportional Cost, QAR
*Fibronectin*			
Positive fibronectin, delivery within 48 hours	0.3112	1,182,124	367,863
Negative fibronectin, delivery within 48 hours	0.6319	1,022,314	646,010
Positive fibronectin, no delivery within 48 hours	0.0336	0	0
Negative fibronectin, no delivery within 48 hours	0.0233	75,980	172.68
*No fibronectin*			
Delivery within 48 hours	0.8084	4,588,393	3,709,084
No delivery within 48 hours	0.1916	3,170,042	607,499
Net saving/per person	QAR 6,470 (US$ 1,777)		
ICER/successful prolongation of delivery within 48 hours, with and without ADRs with fibronectin	Dominant		

**Notes**: fFN probabilities are conditional on the test result (specific result-based pathways), while no-fFN probabilities are overall, cohort-level estimates A cost of zero indicates that no extra hospitalization or treatment costs related to delivery within the modelled time horizon were incurred in this pathway 1 QAR = 0.27 US$.

**Abbreviations**: QAR, Qatari Riyal; ICER, incremental cost-effectiveness ratio; ADRs, adverse drug reaction;, US$, United States Dollar.

When comparing secondary clinical outcomes, significant differences were observed between women who received fFN testing and those who did not. The delivery outside the 48 hours was lower in the fFN group, at 6%, compared to 19.2% in the no-fFN group (p<0.001). While the median time from admission to delivery was similar between the groups (1 day for both, with IQR of 1 day and 2 days, respectively; p=0.83), the median duration of hospitalization during the initial admission was 3 days for fFN and 4 days for no-fFN group, showing a statistically significant difference (p<0.001). Of note, women in the fFN group were more likely to be admitted to the emergency department than those in the no-fFN group (41.1% vs 27.6%, p=0.001). Furthermore, 6.5% of women in the fFN testing group required rehospitalization. All clinical outcomes are summarized in Table 3.

**Table 3:** Clinical Outcomes of Fibronectin and No Fibronectin and Rehospitalization-Related Interventions

Characteristic	Fibronectin (n=232)	No Fibronectin (n=287)	*P value*
Delivery outcome			
Primary outcome (delivery within 48 hours), no. (%)	218 (94)	232 (80)	<0.001
Secondary outcome (outside 48 hours), no. (%)	14 (6)	55 (19.2)	<0.001
Time from admission to delivery, median (IQR), days	1 (1)	1 (2)	0.83
Hospitalization during initial admission, median (IQR), total hospital days	3 (3)	4 (3)	<0.001
Stay in ED, no (%)	n=231	n=286	0.001
Yes	95 (41.1)	79 (27.6)	
No	136 (58.9)	207 (72.4)	
Rehospitalization, no. (%)	n=230	n=287	N/A
Yes	15 (6.5)	Not collected	
No	215 (93.5)	Not collected	
Fibronectin done during rehospitalization			N/A
Yes	0	N/A	
No	232	N/A	
Tocolytic (atosiban) received first cycle during rehospitalization, no. (%)			0.447
Yes	1 (0.4)	0 (0)	
No	231 (99.6)	287 (100)	
Any ADE, no. (%)			N/A
Yes	0 (0)	0 (0)	
No	232 (100)	287 (100)	
Other ADEs, no. (%)			N/A
Yes	0 (0)	0 (0)	
No	232 (100)	287 (100)	

**Abbreviations**: IQR, interquartile range; ED, emergency department; ADE, adverse drug events; N/A, not applicable.

### Sensitivity Analysis

**Figure 2:**
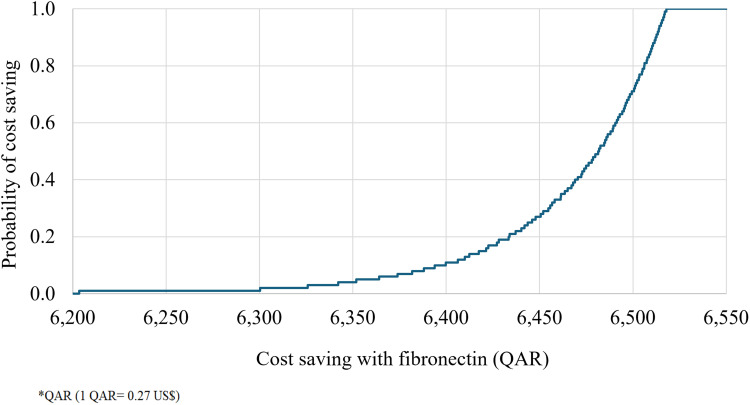
Cost-saving probability curve (one-way sensitivity analysis).

**Figure 3:**
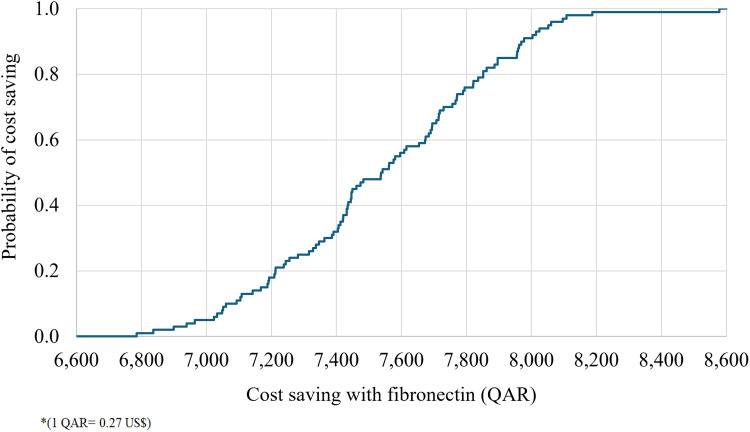
Cost-saving probability curve (Multivariate sensitivity analysis).

Base-case inputs and their uncertainty ranges are reported in Table 4. The model was insensitive to changes in all variables in OWSA and MSA. In the OWSA and MSA, the Monto Carlo simulation illustrated that, with lower cost and higher successful delivery, fFN maintains dominance over no-fFN in 100% of simulated cases (Figures 2 and 3). In the MSA, the mean cost saving of fFN was QAR7,543 (US$2,072), 95% CI QAR 6,785 (US$1,864) to 8,579 (US$2,357) (Table 4).

**Table 4:** Variation Range for Variables Used in Sensitivity Analyses with Outcomes of Interest

One-Way Sensitivity Analysis			
Variable	Base case	Distribution	Cost-Saving Outcome, QAR per Person, Mean 95% CI (Lower, Upper)
Fibronectin acquisition cost	232	Gamma	6,465 (6,204, 6,518)
**Multivariate sensitivity analysis**			
*Cost*			
Fibronectin acquisition cost	232	Gamma	7,543 (6,785, 8,579)
*Probability (fibronectin)*			
Positive fibronectin, delivery within 48 hours	0.3112	Beta	
Negative fibronectin, delivery within 48 hours	0.6319		
Positive fibronectin, no delivery within 48 hours	0.0336		
Negative fibronectin, no delivery within 48 hours	0.0233		
*Probability (no fibronectin)*			
Delivery within 48 hours	0.8084	Beta	
No delivery within 48 hours	0.1916		

**Note**: 1 QAR = 0.27 US$.

**Abbreviations**: QAR, Qatari Riyal; CI, confidence interval.

**Figure 4:**
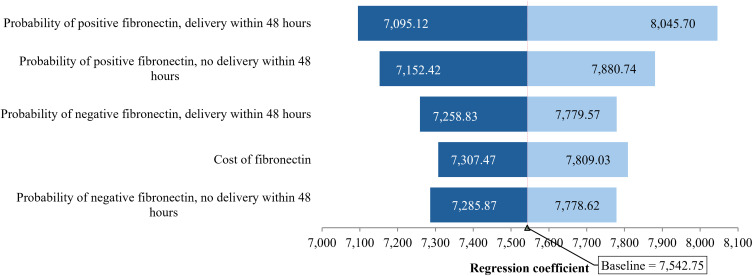
Tornado diagram of outcomes with regression coefficients representing the influence of model parameters on cost outcomes.

A regression tornado diagram is depicted in Figure 4, showing that the pathway probability of positive fFN with delivery within 48 hours and the pathway probability of positive fFN without delivery within 48 hours had the most significant influence on the model outcome. In contrast, the probability of negative fFN without delivery within 48 hours had the least impact on the outcomes.

## Discussion

This study provides valuable insights into the economic and clinical implications of fFN testing in the management of preterm labor in Qatar. In this study involving 519 women, our findings demonstrate that fFN testing is associated with differences in both maternal and neonatal outcomes, as well as substantial cost savings in the Qatari healthcare system. The fFN group achieved a 94% rate of successful delivery within 48 hours compared to 80% in the no fFN group, resulting in a substantial cost saving. Importantly, the higher 48-hour delivery rate seen in the fFN group (94% vs 80%) may in part be due to baseline clinical differences in presentation rather than a direct effect of the fFN test. For instance, variations in gestational characteristics, membrane rupture status, intrapartum bleeding, and other clinical factors may have affected both the decision to conduct fFN testing and the resulting maternal and neonatal outcomes. Consequently, the favorable results seen in the fFN group cannot be attributed to the test alone. Accordingly, the results should be interpreted as associations, not as proof of causality. Also, while the fFN group had a higher birth weight, NICU admission rates were comparable between groups. Thus, any potential advantages of fFN testing may be better reflected in maternal management and short-term healthcare use rather than in more general neonatal outcomes.

Although prior studies have assessed the cost-effectiveness of fFN testing in Europe and North America, data from Middle Eastern healthcare settings are still scarce. Consequently, our study provides Qatar-specific real-world evidence to inform local clinical practice and resource-allocation decisions. To our knowledge, few studies have assessed the cost-effectiveness of fFN testing versus no testing among symptomatic women at risk of preterm birth in Qatar. In our retrospective analysis of women admitted with preterm labor between 2019 and 2022, fFN testing was associated with substantial cost savings and differences in clinical outcomes. These results align with prior studies indicating that fFN testing may improve risk stratification and help optimize healthcare resource use among women presenting with symptoms of preterm labor. Desplanches et al (2018)^8^ evaluated seven diagnostic strategies using a decision-analysis model, whereas our study specifically compared fFN testing with no testing using real-world clinical data from electronic medical records. In addition, their analysis focused on delivery within seven days, while our primary outcome was successful prolongation of pregnancy beyond 48 hours, an interval that is clinically important for administering corticosteroids and providing short-term tocolytic therapy. Although the methodologies differed, both studies suggest that risk-stratification strategies may improve healthcare resource utilization and reduce costs related to preterm labor management. Similarly, Rose et al (2010)^14^ assessed an fFN-based management protocol and reported reductions in hospital admissions and healthcare utilization. However, their study did not include a formal cost-effectiveness analysis and relied on historical comparison groups. In contrast, our study incorporated comprehensive direct medical costs and a formal economic evaluation, offering additional evidence on the potential economic value of fFN testing in routine clinical practice. Moreover, our analysis used more recent data and included detailed clinical and neonatal outcomes derived from standard care. Baaren et al (2017)^24^ also reported that adding fFN testing to management strategies for threatened preterm labor was cost-effective compared with alternative approaches. Nevertheless, their model was based on data from the APOSTEL studies and assessed delivery within seven days, whereas our evaluation used retrospective real-world clinical data and centered on the clinically relevant 48-hour treatment window. Although differences in healthcare systems, patient populations, outcome definitions, and economic modeling approaches limit direct comparability, both studies support the potential role of fFN testing in improving resource allocation for women presenting with symptoms of preterm labor.

Overall, despite differences in methodology and healthcare context, the available evidence generally supports the potential of fFN testing to inform clinical decision-making and optimize healthcare resource utilization. However, the transferability of cost-effectiveness findings across healthcare systems remains limited, underscoring the need for context-specific evaluations, such as the present study.

The implications of our findings cannot be ignored. The demonstrated cost savings of QAR6,470 per case (ie, up to 31% with fFN testing), combined with improved clinical outcomes, suggest that fFN testing could be a valuable element of preterm labor assessment protocols, but prospective studies are required to support definitive recommendations. Such economic evaluations are paramount in an era of rising healthcare costs and limited resources. As healthcare systems globally strive for efficiency, evidence demonstrating the cost-effectiveness of certain diagnostic strategies can inform health policy decisions.

The stochastic nature of healthcare necessitates that any practice recommendations are based on robust data. As already discussed above, the sensitivity analyses performed in this study reinforce the reliability of the findings, asserting that the fFN strategy maintains its status as the most cost-saving approach across different assumptions. This robustness indicates the need for a shift in clinical protocols, advocating for the need to adhere to the fFN testing in routine practice by clinicians. However, while the findings provide a compelling argument for this approach, it is important to also consider other factors such as accessibility, patient adherence to follow-ups, and training of clinicians in implementing fFN testing to maximize their effectiveness. Furthermore, the implications of these findings extend beyond immediate clinical settings. They signify a need for a call to re-assess current guidelines surrounding preterm labor management locally and internationally. Although current international guidelines have not definitively recommended any diagnostic strategy,^19^,^20^ this study presents findings that could prompt regulatory bodies to reconsider their recommendations. Policymakers should also consider not only the clinical and economic effectiveness of fFN testing but also the quality of life for mothers and families affected by preterm labor.^32^ The recent supply disruption and subsequent decision by Hologic to cease production of fFN cassettes have significant implications for the management of preterm labor in the UK,^27^ Australia,^33^ and New Zealand.^33^ In light of these challenges, healthcare providers are now advised to utilize alternative tests, such as Actim^®^ Partus, for women presenting with threatened preterm labor. This situation contrasts with countries like Qatar,^32^ the US,^34^ and many European nations,^35^ where fFN testing remains available and continues to be a central component of preterm labor assessment protocols. The ongoing use of fFN testing in these countries not only informs clinical practice but also contributes to favorable maternal and neonatal outcomes, allowing for targeted interventions and reduced healthcare costs.

Importantly, a study by Bruijn et al,^36^ demonstrates that the Actim^®^ Partus test can serve as a valuable alternative, particularly when used in conjunction with cervical length measurement In their post-hoc analysis of a nationwide cohort study involving women with signs of preterm labor, combining the Actim^®^ Partus test with cervical length measurement yielded high sensitivity (91%) and negative predictive values (97%). These findings suggest that the Actim^®^ Partus test can effectively identify women who are at low risk for preterm delivery, making it a suitable alternative to the fFN test in environments where access to fFN testing is limited.

Policymakers should consider the efficacy and cost-effectiveness of both the fFN and Actim^®^ Partus tests, given their implications for clinical outcomes amid accessibility challenges and varying resource availability in different healthcare settings. Furthermore, collaboration among international health organizations could promote the sharing of best practices and insights into optimizing preterm labor management despite potential supply issues, ensuring that all pregnant women receive the most effective care regardless of their geographical location.

Also, the challenge of managing preterm labor includes not only clinical considerations but also socio-economic factors that can determine the level of care available to women. The comprehensive evaluation of diagnostic strategies illustrated in our study provides a framework through which clinicians can make informed choices that optimize both maternal and neonatal outcomes. Given the substantial impact of preterm birth on a child’s health, including long-term developmental challenges and increased healthcare needs, the economic implications have far-reaching consequences. The reduction of neonatal morbidity and mortality not only has immediate benefits but also enhances the potential long-term costs associated with ongoing medical care for conditions arising from preterm birth. Thus, future research should explore the long-term effects of fFN testing and evaluate its integration into standardized care protocols across diverse healthcare settings. Although not evaluated in the current study, emerging predictive technologies may further improve risk stratification in preterm labor and warrant future investigation.^37^,^38^ The main strength of our study is that it was based on real-world data from electronic medical records Cerner. As was also implied earlier, we included a long-term outcome of neonates, following up the neonates until their NICU discharge. However, there are limitations in the current study. The retrospective design may introduce biases related to data collection and patient selection, potentially affecting the internal validity of our results. Also, because the study was retrospective and observational, causal conclusions cannot be firmly drawn. The decision to perform fFN testing was based on clinician judgment rather than random assignment. Therefore, women in the fFN and no-fFN groups may have differed in clinical severity and risk profile, introducing selection and indication bias. As a result, between-group differences may reflect underlying clinical characteristics or clinician decision-making rather than the direct effect of fibronectin testing. In particular, baseline disparities, such as membrane rupture status and gestational characteristics, suggest that the groups may not be directly comparable. Furthermore, women who received fFN testing may have differed in important respects from those who did not, and clinical management decisions (eg, admission and treatment) may have been influenced by factors such as disease severity and physician preferences, further contributing to selection and indication bias. Additionally, the analysis focused on short-term outcomes, specifically delivery within 48 hours, and did not assess longer-term neonatal sequelae, long-term healthcare utilization, or related downstream costs that may result from preterm birth. The economic analysis also considered only direct medical costs, which may underestimate the overall economic burden. In particular, indirect costs such as productivity losses, caregiver burden, and broader societal costs were not included; therefore, the total impact of preterm birth and its management may be understated. Moreover, no multivariable adjustment or propensity score matching was performed, so residual confounding due to both measured and unmeasured baseline differences cannot be ruled out. Although one-way and probabilistic sensitivity analyses were conducted to explore parameter uncertainty, structural uncertainty related to model design, assumptions, and the selected time horizon was not formally assessed. Accordingly, the results should be interpreted in light of the underlying model structure. Additionally, while we reported substantial cost savings associated with fFN testing, the lack of long-term follow-up data, beyond the site, restricts our ability to investigate its comprehensive impact on maternal and neonatal health outcomes. Finally, because this study was carried out in a single tertiary care center in Qatar, the results may not be completely generalizable to other healthcare settings with different patient populations, clinical approaches, and patterns of resource use. Another limitation concerns the strategies modelled. Here, despite the fact that we considered the use of new diagnostic tests such as quantitative fFN in our study, the combination of quantitative fFN and cervical length was not included in the model due to a lack of high-quality data.^39^ In addition, in relation to the economic analysis, only direct medical costs were considered, which may underestimate the potential lost productivity cost in included women.

## Conclusion

Our study suggests that fibronectin testing may be associated with better short-term clinical outcomes and lower healthcare costs for women presenting with preterm labor. These results outline the potential role of fFN testing in helping guide clinical decision-making and resource utilization in managing preterm labor. However, because the study was observational and the groups differed at baseline, along with the possibility of residual confounding, the, the results should be interpreted cautiously. Prospective multicenter studies isare needed to assess the long-term neonatal outcomes, indirect costs, and the overall clinical and economic benefits of fibronectin testing.

## Data Availability

The data that supports the findings of this study are available in the supplementary material of this article.
